# Anxiety and Depression in Patients with Aseptic Osteonecrosis at Different Skeletal Sites: A Cross-Sectional Study”

**DOI:** 10.1192/j.eurpsy.2026.11229

**Published:** 2026-07-31

**Authors:** A. Feki, I. Sallemi, A. Abbes, Z. Gassara, H. Fourati

**Affiliations:** 1 Rheumatology Department; 2 Occupational Medicine Department, Hedi Chaker Hospital, Sfax, Tunisia

## Abstract

**Introduction:**

Avascular necrosis, also known as aseptic osteonecrosis (ON), is a progressive disorder brought on by compromised bone vascularisation. ON may affect the knee, shoulder, ankle, and other skeletal areas, while the femoral head is the most often impacted area. Chronic pain, impairment, and loss of function are the results of this condition. Although ON may have an impact on prognosis and quality of life, less is known about its psychological effects, especially those related to anxiety and depression.

**Objectives:**

To evaluate the prevalence and severity of anxiety and depression in patients with aseptic osteonecrosis at different skeletal sites and to explore associated clinical factors.

**Methods:**

In a cross-sectional analysis, we included patients from a tertiary rheumatology service who had been diagnosed with aseptic osteonecrosis (hip, knee, shoulder, ankle, and multifocal locations) from 1995 to 2024. Data on radiography, demographics, and clinical conditions were gathered. The Visual Analogue Scale (VAS) was used to measure pain, and site-specific scores were used to assess functional restriction. The Hospital Anxiety and Depression Scale (HADS) was used to evaluate anxiety and depression. Correlations with illness location, stage of ON according to the classification of Arlet and Ficat, and pain were investigated statistically.

**Results:**

Among 43 patients (mean age 53 ± 11 years, 48.8% male), osteonecrosis involved the hip (30 cases, 69.7%), knee (5 cases, 11.6%), shoulder (4 cases, 9.3%), semilunar (3 cases, 6.9%) and multifocal sites (1 case).

The diagnosis of osteonecrosis was confirmed by simple radiography in 15 cases, bone scan in 14 cases and magnetic resonance imagery in 14 cases. The median VAS was 7, and the interquartile range (IQR) was 6 to 8.

Clinically significant anxiety and depression were detected in 42% and 38% of individuals, respectively.

Psychological distress was more common in patients in later stages and those with higher assessments of pain (p<0.01).

**Conclusions:**

Regardless of the skeletal location, anxiety and depression are common comorbidities in individuals with osteonecrosis. Multidisciplinary treatment and systematic psychological assessment are crucial for bettering overall patient outcomes and management.

**Disclosure of Interest:**

None Declared

